# Chicago Medical Society

**Published:** 1886-08

**Authors:** 


Officially Reported for The Atlanta Medical and Surgical Journal.

### CHICAGO MEDICAL SOCIETY.

Stated Meeting, June 7, 1886.

The President, E. L. Doering, M. D., in the chair.

Dr. A. Reeves Jackson read a paper entitled

THE INTRA-UTERINE STEM IN THE TREATMENT OF FLEXIONS, exhibiting the stems used.

The essayist began treating uterine flexions with the stem pessary in 1870. Prior to that time the only methods he had employed were gradual dilatation and incisions. The results were so unsatisfactory that he sought for a safer and more successful method. Having received the impression that the use of the stem pessary was more hazardous than either the dilating or cutting plans, he commenced its employment with misgiving, and did not rely wholly upon it, but preceded it with either gradual stretching or slight incisions. In two cases this mixed method was followed by pelvic abscess, a sequence which he had never observed when the stem alone had been used. All cases of uterine flexion are not accompanied by dysmenorrhœa or sterility, yet when there exists a relationship between these symptoms and an existing flexion, the latter must be looked upon as a mischievous factor, and one that should be removed. He had never treated any case of flexion in which dysmenorrhœa was not present, although co-existent barrenness has been frequently an additional incentive to the patient to undergo efforts at cure.

He preferred Chambers' bifurcated vulcanite instrument, although the divergence of the branches *below* the internal os uteri was a radical defect in the instrument as ordinarily used. Frequently the branches should be closed so that the stem might be practically single in that portion which traverses the cervix. His method is as follows : A flexion and its direction being diagnosticated, a flexible bougie is passed through the bent portion of the canal and quite to the fundus. The depth of the canal being carefully noted, a pliable stem, consisting of the distal portion of the same, or a similar bougie, one-third of an inch shorter than the ascertained depth of the canal, is selected for introduction. A flange or bulb is formed upon the outer end of the stem by rolling upon it a section of rubber tubing. The woman being placed on the back in Simon's position and the os uteri exposed with a speculum, the stem, either grasped with a dressing-forceps or mounted upon the end of a piece of pointed wire, is passed entirely into the uterus. A large tampon of cotton moistened with slightly alumized glycerine is pressed against the bulb of the stem, and allowed to remain one or two days. The tampon is removed and replaced at suitable intervals until the tendency of the stem to leave its position disappears. After this yielding stem has remained from one to three weeks, according to the degree of tolerance manifested by the uterus, it is removed and a thicker one put in its place. This likewise is permitted to remain a week or two, and is then replaced by a Chambers stem. While not very much, or, indeed, any change of shape is to be expected in consequence of the use of the flexible stem, yet, in several instances, a very considerable alteration took place within a few weeks, or even a few days, and in a few cases it was found unnecessary to resort to a rigid instrument at all. Usually, however, it had been found necessary to use an inflexible instrument for from three months to a year—not continuously, but for periods of three or four months, with an interval of a week or two, during which the stem was removed in order to test the degree and permanence of the improvement. The feature of this treatment which is essential to its safety and success is its slow and gradual conduct, and the non-observance of this necessity has been the cause of dangerous results and failures to cure.

The drawbacks attending this method of treatment were: 1. Difficulty in retaining the instrument in position; 2. Pain; 3. Hemorrhage; 4. Pelvic inflammation—all except the first being common to all other methods of treatment. A table, comprising the details of sixty-four cases treated by the intra-uterine stem alone, was given, showing the ages and social conditions of the patients, the direction of the flexion and the result of the treatment. Of the entire number 42 occurred in married and 22 in single women. Of the former 8 had borne children; the other 34 were sterile. Of the latter 8 subsequently bore children. A cure of the flexion followed in 40; of the remaining 24 four were improved and relieved of dysmenorrhœa. In 20 the result was unknown. The ages of the patients ranged from 19 to 39 years. The uterus was anteflexed in 50 and retroflexed in 14.

In conclusion, the author said: "I believe the principle of the intra-uterine stem in the treatment of flexions to be correct, and it need not be dangerous—at least, no more dangerous than any other effective method. I further believe that by its use more cases of uterine flexion can be cured than by any other means at present in vogue. The conditions of both safety and success are watchfulness, patience, and slow progress."

Dr. D. T. Nelson, in opening the discussion, said: I am glad to have heard the paper, and think it is a most valuable one. The cautions that it gives are certainly those that all of us should remember, to-wit, the length of the instrument used compared with the length of the uterus, the slow and gradual dilatation of the uterus before using the inflexible stem, and removing it on the occurrence of bad symptoms. In recent years I have not been in the habit of using the stem pessary as much as my friend, Dr. Jackson, but I think that, with his present instructions, I shall try it again. Not that I have not tried gradual dilatation, and the gradual, slow, careful straightening of the uterus, but I have not by this particular means caused the pessary to be retained as constantly as he has. The vulcanite pessary, and the various other forms, including the Wright's or Chambers' modification, I have used and with many of the difficulties the doctor has narrated. But with his modification it seems to me very likely we can use them with better success. The irritation produced by them has been a great drawback, and in recent years it has been my habit rather to use the form of pessary recommended by one of our members, Dr. W. H. Byford, the slippery elm bougie. It produces a gradual dilatation of the uterus, and often produces remarkable results in the treatment of the flexions, and I have had no bad results from its use.

Dr. T. D. Fitch said: I think a paper so commendatory of a measure as this will perhaps lead many of the members of the profession to adopt it without proper precautions and without realizing the dangers which attend the use of the intra-uterine stem. I believe it is a very dangerous instrument to use. I am an advocate, as you all know, of pessaries, but I do think the intra-uterine stem a dangerous instrument, and that in less careful hands than Dr. Jackson's serious results will often follow.

Dr. A. C. Feeder said: Much depends upon the state of the patients at the time of treatment, whether they are in a healthy condition, or whether they have some specific blood disease in which medicine would assist in the treatment. And if the medicine has an alterative effect, how much benefit is received from the medicine and how much from the pessary.

Dr. H. P. Merriman said: I have very little to add to what has been said. The use of the various methods that have been proposed seem to me to aim at one given end—to change the nutrition of the uterus. Forcible dilatation does that to a certain extent; it is temporary, however, in its action. Incision produces an alterative effect and accomplishes its purposes. It does not succeed a great many times, neither does the temporary action of dilatation. The use of the stem pessary, on the other hand, succeeds because it is keeping up a continuous pressure upon the parts. Now I am decidedly in favor of this treatment by stem pessary; it strikes me that it is the only rational method of treating these flexions, which are pathological. After the cause of a flexion has been removed, that is, the inflammation of the uterus or the pressure of a tumor, or pressure of heavy clothing, or whatsoever causes it, the uterus does not always return to its natural state, and then we need to introduce some method for restoring it to its normal condition, and I do not know any more rational method than this one.

Dr. Sarah H. Stevenson said: I have listened to the paper with a great deal of interest, and also to the discussion. My methods are different; I have used the stem pessary a great deal in former years, but for the past two years I have discarded it entirely, as some of the results were unfortunate, although I think I have never had any serious results from the use of the stem.

Dr. H. T. Byford said: I congratulate Dr. Jackson that he has discarded incision and dilatation in treating flexions. I also congratulate him upon his good success. I believe the mortality from this treatment—the treatment by the intra-uterine stem—has been estimated to be from one-half to one per cent. by those who have investigated heretofore. Whether it is so now I do not know. The present per cent. of inflammation of the cellular tissue varies from two to five per cent. as nearly as I can determine. There are an immense number of cases in which the stem caused inflammation, which have never been published. It seems to me that in considering this subject the reason for this treatment should be made more apparent. There are some who use it as a splint or merely to straighten the uterus; others use it as a stimulant on account of its continuous pressure. There is no doubt it stimulates and temporarily straightens the uterus, but it is well known that in time, in a large proportion of these cases, the uterus again becomes flexed.

Dr. E. C. Dudley said: Mr. President: The marvelous freedom from dangerous inflammation following the treatment of uterine flexure by forcible dilatation, and by the application of the intra-uterine stem, furnishes a striking illustration of the fact that the human uterus will sometimes endure an immense amount of abuse. My own preference is generally for the former method, as advocated by Goodell, Ellinger and others. My experience has only tended to confirm me in the impression that forcible dilatation is reasonably satisfactory in its results, and that the results are reasonably permanent. I would seldom advocate the use of intra-uterine stem pessaries for retroflexion unless the flexure were of the so-called congenital variety, and therefore associated with atrophy of the uterus, a condition which is very rare.

Dr. Jackson said in concluding the discussion: Mr. President, I feel that I ought to express my thanks for the courtesy with which this paper has been received. It is only a thirteen minute paper, and there are a great many things in the domain of medicine that are not in it, and a great many questions might be asked on subjects growing out of and connected with it, which I could not answer if I were disposed to. The intention of the paper is simply to demonstrate the efficiency of a single remedy in correcting a single deformity. Questions as to whether the uterus was prolapsed, whether the patients had taken anti-bilious pills, or had cachexia, really do not enter into the consideration of the subject. I supposed that was perfectly plain from the fact that no mention was made of anything beyond the mere condition of deformity. I am very glad so many excellent ideas have been added to it. The suggestion of Dr. Nelson as to the patient being able to withdraw the pessary is excellent and is never omitted. I never introduce a pessary that I do not attach a silken cord by means of which the patient can withdraw it in case of necessity. As a rule, every patient should be able to withdraw any instrument placed in the genital passages; the regular attendant may not be at hand when needed; there may be an aversion on the part of the patient to calling in another physician, and she should have the proper means at her disposal. The suggestions made in the discussion accounting for the safety and success of the treatment are, I think, correctly attributed to the preliminary measures—the slowness of the straightening and the promptness with which any tendency to harm could be met. The object was to accustom the uterus to its tenant, so that by and by it would accommodate a larger one, and in this way the uterus has been made to receive and tolerate the presence of an inflexible instrument. In one case a Chambers' stem was retained twenty months, and I think if the patient had not returned and told me she was wearing it, it would be there yet. It produced no unfavorable symptoms.

Dr. A. R. Small reported

A CASE OF PISTOL-SHOT WOUND.

May 2d, 1886, he was called to see F. R., aged 23, who, a few minutes previously, had received a shot from a No. 32 pistol. Patient was suffering from shock, difficult breathing, and excessive pain in the left leg below the knee. The ball had struck the right eighth rib, about two inches external to the costal cartilage. Sensation was lost in the right leg below the knee. Motion was not impaired in the right leg, though the sensation was lost below the knee. The left leg was hyperæsthetic below the knee and motion slightly impaired. A drainage-tube was inserted about two inches into the wound, and the wound dressed antiseptically. The patient complained of no pain except in left leg below the knee, where the pain was excessive. Morphia was given hypodermically in sufficient doses to control the pain. Nothing was allowed the patient the first twelve hours but ice and occasionally water. About 10 p. m. there was evidence of internal hæmorrhage, and the patient seemed to be sinking. Milk was then given in small quantities frequently. The morning of the 3d he had rallied somewhat.

The urine was drawn by the catheter every eight hours and contained blood. There was no expulsive force to the bladder. Respiration was normal after the first two hours.

On the afternoon of the third, patient became delirious, and continued so, with occasional lucid intervals, until death, which occurred at 4:20 p. m. of May 4th.

Autopsy five hours after death. Rigor mortis well marked. Unfortunately, through a misunderstanding, the undertaker had preceded us and injected his preserving fluid, so that we were unable to determine exactly the amount of blood in the right pleural cavity. It must have been quite large, however, as the right lung was entirely collapsed. The ball made a clean round hole through the center of the eighth rib on the right side, about two inches from the costal cartilage, passed through the lower side of the right pleural cavity, without injuring the lung, passed through the diaphragm, right lobe of the liver,and superior portion of the right kidney, and through the intervertebral foramen between the eleventh and twelfth dorsal vertebræ, on the right side of the spine, and lodged against the posterior surface of the body of the eleventh dorsal vertebræ, just within the spinal cord, where it was so firmly imbedded that it could not be removed without disarticulating the spine, which, for sufficient reasons, we did not do.

Though we found the right lung collapsed, respiration had been normal except the first two hours after injury.

Dr. Alfred S. Houghton read a paper on

THE DANGER IN SPECIALISM.

He said that specialties had greatly increased medical knowledge and skill, and had secured for many much reputation. Hence young practitioners grasp any excuse for becoming specialists. But man is not a machine, but a complicated organism, and disease is complex, one organ sympathing with another; hence it is necessary to examine every portion of the body and treat all organs affected. Hence there is danger in a specialist limiting his sphere of action and usefulness to an unnecessary degree. Another danger is that specialists are apt to become egotistic, and give rise to utterances which they will afterwards regret.

